# Supplementary material for: Neural network identification of people hidden from view with a single-pixel, single-photon detector
Source: Sci Rep. 2018 Aug 9;8:11945. doi: 10.1038/s41598-018-30390-0 (PMC6085360; doi:10.1038/s41598-018-30390-0)
Supplement: Supplementary file 1 — Supplementary Information [file 41598_2018_30390_MOESM1_ESM.pdf]

SUPPLEMENTARY MATERIALS: Neural network identification of people hidden from view with a single-pixel, single-photon detector

Piergiorgio Caramazza[1,2], Alessandro Boccolini[1], Daniel Buschek[3], Matthias Hullin[4], Catherine F. Higham[5], Robert Henderson[6], Roderick Murray-Smith[5] and Daniele Faccio[2,*]*

[1]*Institute of Photonics and Quantum Sciences, Heriot-Watt University, Edinburgh EH14 4AS, UK*

[2]*School of Physics and Astronomy, Kelvin Building, University of Glasgow, Glasgow G12 8QQ, UK*

[3]*Media Informatics Group, University of Munich (LMU), Munich, Germany*

[4]*Institute for Computer Science II, University of Bonn, Friedrich-Ebert-Allee 144 53113 Bonn, Germany*

[5]*School of Computing Science, University of Glasgow, Glasgow G12 8QQ, UK and*

[6]*School of Engineering, Institute for Integrated Micro and Nano Systems, University of Edinburgh, Edinburgh EH9 3JL, UK*

---

* *Daniele.Faccio@glasgow.ac.uk

I. NEURAL NETWORK CLASSIFIER.

We build a nonlinear classifier with the aim of correctly identifying the label of histograms resulting from the acquisition of pulsed laser light backscattered from three different people in seven different positions. We use a supervised approach where the input vector (a temporal histogram) is paired to an output vector encoding the class of the person and the target location. Both class of person and location are treated as categorical classification tasks, and encoded using a 'one-hot' encoding with N_c binary outputs for the person classes and N_l binary outputs for location positions. In this work $N_c = 3, N_l = 7$. The cost function, minimised during learning, is the categorical cross-entropy loss [1]. For the i-th observation, the cross-entropy loss of the person class is

$$CCE_{c,i} = \sum_{j=1}^{N_c} \left(y_{c,i,j} \ln o_{c,i,j} + (1 - y_{c,i,j}) \ln(1 - o_{c,i,j}) \right),$$

where $o_{c,i,j}$ and $y_{c,i,j}$ denote the predicted class and the true class for the j-th person respectively. Similarly for location, the cross-entropy loss for location is

$$CCE_{l,i} = \sum_{k=1}^{N_l} \left(y_{l,i,k} \ln o_{l,i,k} + (1 - y_{l,i,k}) \ln(1 - o_{l,i,k}) \right),$$

where $o_{l,i,k}$ and $y_{l,i,k}$ denote the predicted location and the true class for the k-th location respectively. As we are classifying simultaneously both person and position, the resulting cost function is the joint effect of the two cost functions on the person and location output vectors. The cost, L, minimised over the whole training set of N examples is

$$L = -\frac{1}{N} \sum_{i=1}^{N} \left(CCE_{c,i} + CCE_{l,i} \right).$$

The ANN architecture processes input data in parallel through: a fully-connected layer in order to retrieve more information about the distance; and convolutional block layers which, due to their translation invariant nature, focus more on the temporal histogram shape and features. After a number of layers, the output layer comprises two groups of softmax sublayers, associated with person class and location respectively. The largest architecture tested is shown in Figure 1. We used the flexible, open-source Keras library to implement our ANN in Python [1], for an example of how the code looks see Section II. The network weights were regularised using l_2 weight decay with a constant of 0.001. To prevent over-fitting and to encourage generalisation, a dropout layer was used after each dense or fully connected layer followed by a batch normalisation layer. In the convolutional blocks, the convolutional layer is normalised (using a batch normalisation layer) and

down sampled (using a max-pooling layer) except for the last block. For the first two blocks, the convolutional filter size is 10×1 and for the last two blocks this is reduced to 5×1. One hundred such filters are applied in each block. The optimisation algorithm was stochastic gradient descent, with a learning rate of 0.001, Nesterov momentum [2] and was applied for at least 100 iterations.

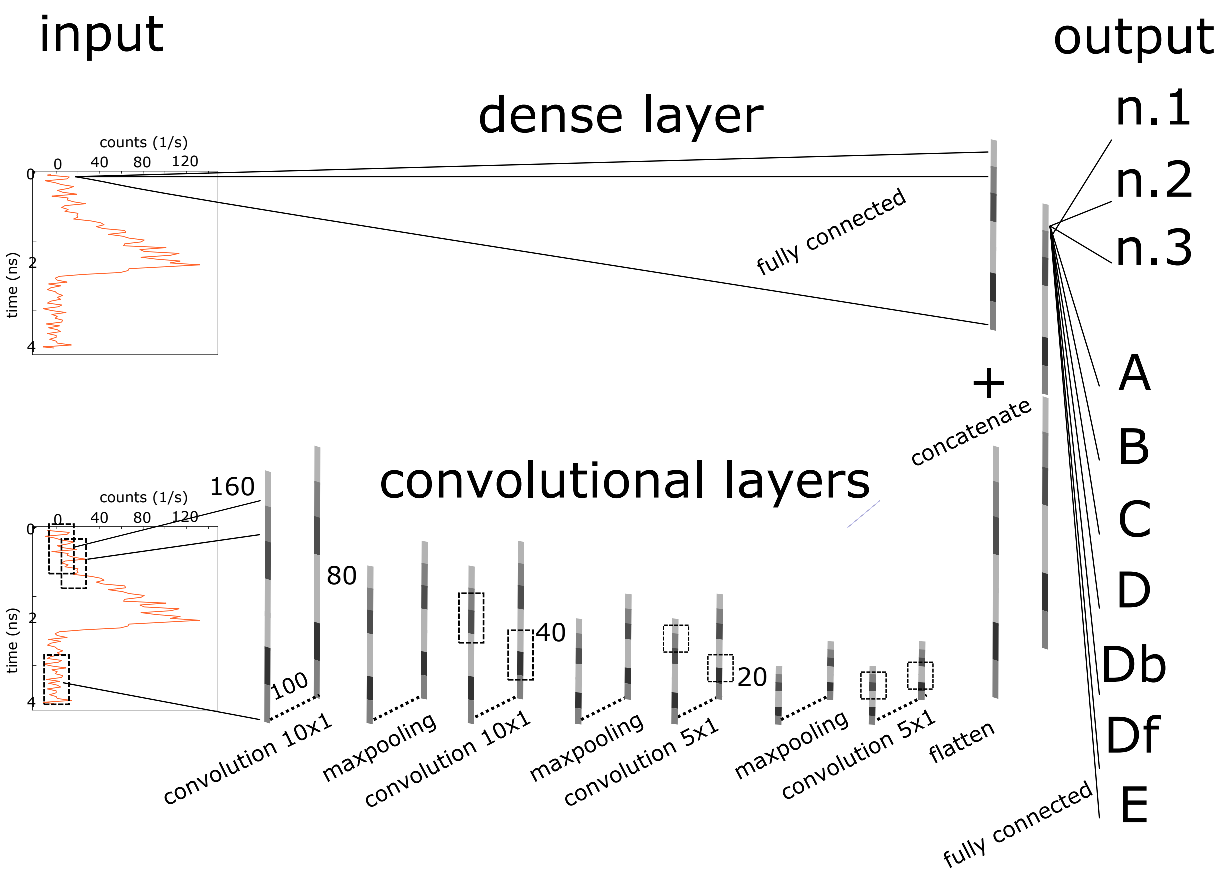

FIG. 1. **The ANN architecture.** The temporal histogram for one pixel forms the input vector for two parallel layers. The upper layer is a fully connected layer (each input component is connected to each network neuron) that extracts information across the pixel profile. The lower layers comprise convolutional blocks which are designed to focus on the shape-like features of the profile by passing small one dimensional filters over the input. The output from each parallel layer is concatenated and connected to 3 person (n.1, n.2 and n.3) and 7 location (A, B, C, D, Db, Df and E) classes. The softmax function is applied to the final layer and the network is trained under categorical cross-entropy loss.

A second architecture was tested, which differs from the first architecture primarily by reducing the number of filters from 100 to 32 for the first two blocks and 64 for the last two blocks. The convolutional filter size is 5×1 for all blocks. Average-pooling replaces max-pooling in the second and third block and batch-normalisation is removed from the convolutional blocks. A noisy input variant to each architecture was also tested. In these variants, a customised additive Gaussian noise

layer re-introduces background noise to encourage generalisation. However, the results suggest that the form and level of the noise, based on just two day's readings, confound rather than improve the results.

Finally, we compare the results from different ANN architectures in Fig. 2 by reporting the correct-prediction percentages for each individual class (i.e. the diagonal values in the confusion matrices). The first algorithm (a) refers to the results presented above. In (b) a noise layer has been added in order to help the network generalise robustly to variation associated with noise in the sensor. The convolutional side is simplified in architecture (c), primarily by reducing the number of convolutional filters. A noise layer was added to (c) in the architecture (d). Another further simplification is applied for architecture (e) where only the fully-connected layer was considered. In (f), a noise layer is added to (e) as well. Finally, we classify separately both people and positions with just the fully-connected layer in (g). As we can observe, the results tend to not show any particular sensitivity to the specific ANN architecture employed, therefore suggesting the robustness of this modelling approach and that further improvement would need to come from larger or more controlled training sets. However, the ANN performance does seem to suggest that the approach of classifying location and identities jointly is consistently better than trying to deal with these individually. This is probably because the internal representations learned to predict class can then be useful to help predict location more accurately, and vice versa.

An alternative approach to evaluation of the test results can be taken. Instead of taking average classification performance at a per-pixel level, we can base a classification on a majority verdict, all the *ca* 800 per-pixel classifications for a single measurement and average performance at this level. This can then be repeated in the same cross-validation manner used earlier. The goal here is both that it illustrates how we can potentially improve performance by integrating over multiple classifiers, and can also highlight whether the variability in the training set is mostly within pixels (probably relating to the sensor itself) or within measurements (relating to variations in light, pose, movement or clothing). The results of this approach indicate that misclassification is high within certain measurements (typically one out of five measurements collected for a specific person-location), see Figure 3. This suggests that possible differences between measurements are not fully captured in the training data and are reducing the ability of the classifier to interpret previously unseen variations in the test data. Low variation in the data may also explain why the architectural changes were inconclusive. Increasing the variation within the training data should improve the classifier and these findings will inform future directions for this work.

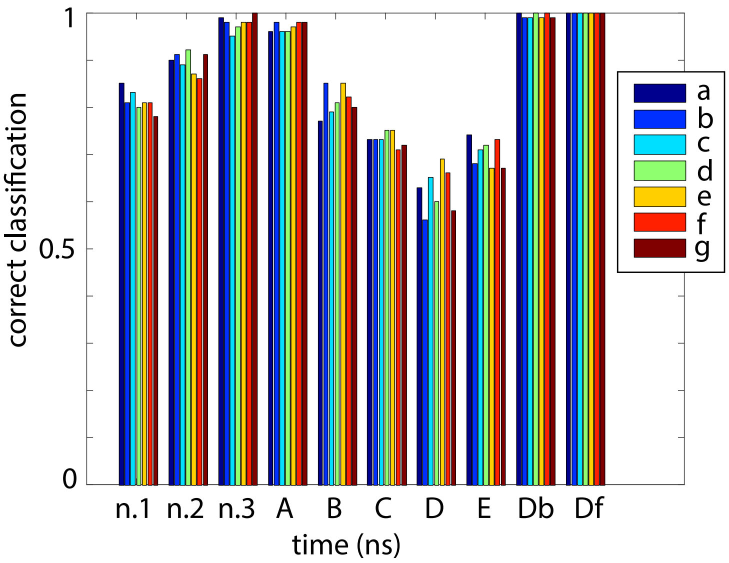

FIG. 2. A comparison between different architectures for the "same clothing" case is reported by showing the individual correct classification for each employed class. (a) A fully-connected layer and convolutional layers in parallel, (b) as (a) but with a background noise layer added after the input, (c) a fully-connected layer and simplified convolutional layers in parallel, (d) as (c) but with a background noise layer added after the input, (e) a fully-connected layer only, (f) a fully-connected layer and a background noise layer and (g) a fully-connected layer for only people and a fully-connected layer for only location.

II. REFERENCES

[1] Francois Chollet. Keras. `https://github.com/fchollet/keras`, 2015.

[2] Ilya Sutskever, James Martens, George Dahl, and Geoffrey Hinton. On the importance of initialization and momentum in deep learning. In *International conference on machine learning*, pages 1139–1147, 2013.

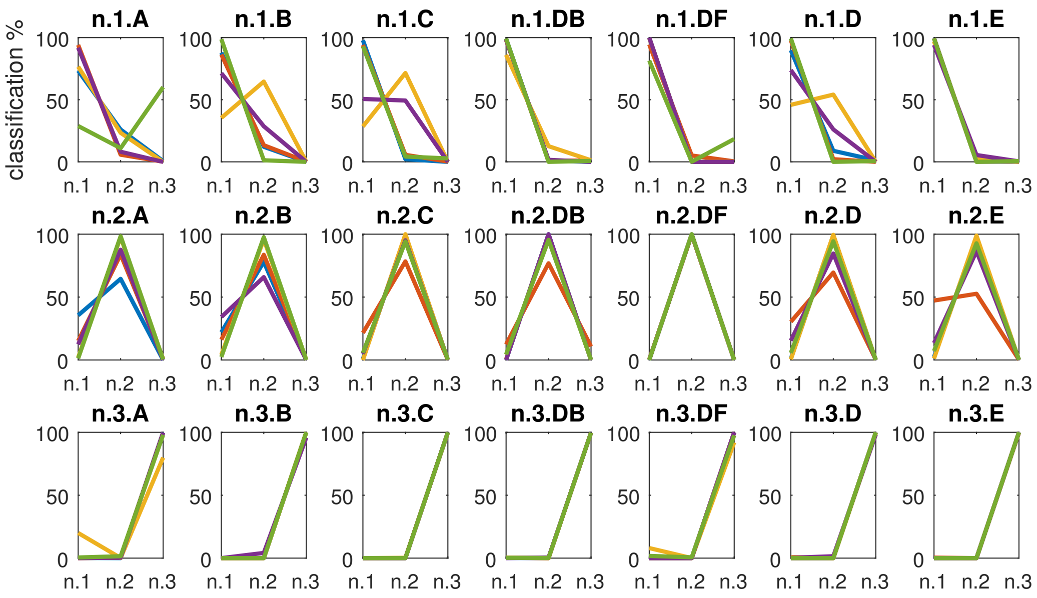

FIG. 3. **Person Classification (% correct) results broken down by measurements.** Classification (% correct) is shown for each person (n.1 *top row*, n.2 *middle row* and n.3 *bottom row*) and each location (A *first column*, B *second column*, C *third column*, Db *fourth column*, Df *fifth column*, D *sixth column* and E *seventh column*) and for each of five measurements (*blue*, *red*, *yellow*, *purple* and *green* lines). For correct classification of n.1, the line peaks should lie over the n.1 *x-axis* position on the *top row*. Likewise for correct classification of n.2 on the *middle row* and correct classification of n.3 on the *bottom row*, the line peaks should lie over the n.2 *x-axis* position on the *middle row* and the n.3 *x-axis* position on the *bottom row*, respectively. For most person-location pairs, the lines are in agreement but for some, *e.g.* n.1.A, one line differs indicating misclassification within this measurement.

III. EXAMPLE: PYTHON KERAS SCRIPT FOR THE ANN MODEL.

```
def setup_model_default(units, X_TR, l2_value, useConv, onlyClass, Ntr, ep_value,
                        y_TRonehot, X_TE, y_TEonehot, y_TRLonehot, y_TELonehot):

# X_TR and X_TE are the training and testing temporal histograms respectively.
# y_TRonehot and y_TEonehot are the person labels for each histogram respectively.
# y_TRLonehot and y_TELonehot are the location labels for each histogram respectively.
# l2_value is the l2 weight decay constant.
# Ntr is the number of training inputs.
# ep_value is the number of training epochs.

    print('Model: Default')

    regConst   = l2_value
    input_vec  = Input(shape=(X_TR.shape[1],))
    input_vec2 = ExpandInput()(input_vec)

    xd = Dense(units, kernel_regularizer=l2(regConst),input_shape=(X_TR.shape[1],))(input_vec)
    xd = BatchNormalization()(xd)
    x  = Dropout(0.5)(xd)
    xd = Activation('relu')(xd)

    if useConv:
        x = Convolution1D(units, 10, padding='same', kernel_regularizer=l2(regConst),
                          input_shape = (X_TR.shape[0],X_TR.shape[1],1))(input_vec2)
        x = BatchNormalization()(x)
        x = Activation('relu')(x)
        x = MaxPooling1D()(x)

        x = Convolution1D(units, 10, padding='same', kernel_regularizer=l2(regConst))(x)
        x = BatchNormalization()(x)
        x = Activation('relu')(x)
        x = MaxPooling1D()(x)

        x = Convolution1D(units, 5, padding='same',activation='relu', kernel_regularizer=l2(regConst))(x)
        x = BatchNormalization()(x)
        x = Activation('relu')(x)
        x = MaxPooling1D()(x)

        x = Convolution1D(units, 5, padding='same',activation='relu', kernel_regularizer=l2(regConst))(x)
        x = BatchNormalization()(x)
        x = Activation('relu')(x)

        x = Flatten()(x)
        x = layers.concatenate([x, xd], axis=1)
    else:
        x = xd

    x = Dense(units, kernel_regularizer=l2(regConst))(x)
    x = Dropout(0.3)(x)
    x = BatchNormalization()(x)
    x = Activation('relu')(x)

    x = Dense(units, kernel_regularizer=l2(regConst))(x)
    x = Dropout(0.2)(x)
    x = BatchNormalization()(x)
    x1 = Activation('relu')(x)
```

```
xC = Dense(3,activation='softmax', kernel_regularizer=l2(regConst))(x1)
xL = Dense(7,activation='softmax', kernel_regularizer=l2(regConst))(x1)

if onlyClass:
   model = Model(input_vec, outputs=xC)
else:
   model = Model(input_vec, outputs=[xC,xL])

# randomly change order of inputs.
order= np.random.permutation(Ntr)
# train the model using SGD + momentum.
batch_size     = 32
nb_epoch       = ep_value
learning_rate = 0.001
decay_rate     = 0.0
sgd = SGD(lr=learning_rate, decay=decay_rate , momentum=0.9, nesterov=True)
if onlyClass:
   model.compile(loss='categorical_crossentropy', metrics=['accuracy'], optimizer=sgd)
   hist_conv = model.fit(x=X_TR[order,:],
                         y=y_TRonehot[order,:],
                         batch_size=batch_size, epochs=nb_epoch,
                         validation_data=(X_TE, y_TEonehot), verbose=2)
else:
   model.compile(loss=['categorical_crossentropy','categorical_crossentropy'],
                 metrics=['accuracy'], optimizer=sgd)
   hist_conv = model.fit(x=X_TR[order,:],
                         y=[y_TRonehot[order,:],y_TRLonehot[order,:]],
                         batch_size=batch_size, epochs=nb_epoch,
                         validation_data=(X_TE, [y_TEonehot,y_TELonehot]), verbose=2)

return model, hist_conv
```
